# Supplementary figures and images for: Integrative Analysis of Transcriptome and Metabolome Reveals the Pivotal Role of the NAM Family Genes in Oncidium hybridum Lodd. Pseudobulb Growth
Source: Int J Mol Sci. 2024 Sep 26;25(19):10355. doi: 10.3390/ijms251910355 (PMC11476975; doi:10.3390/ijms251910355)

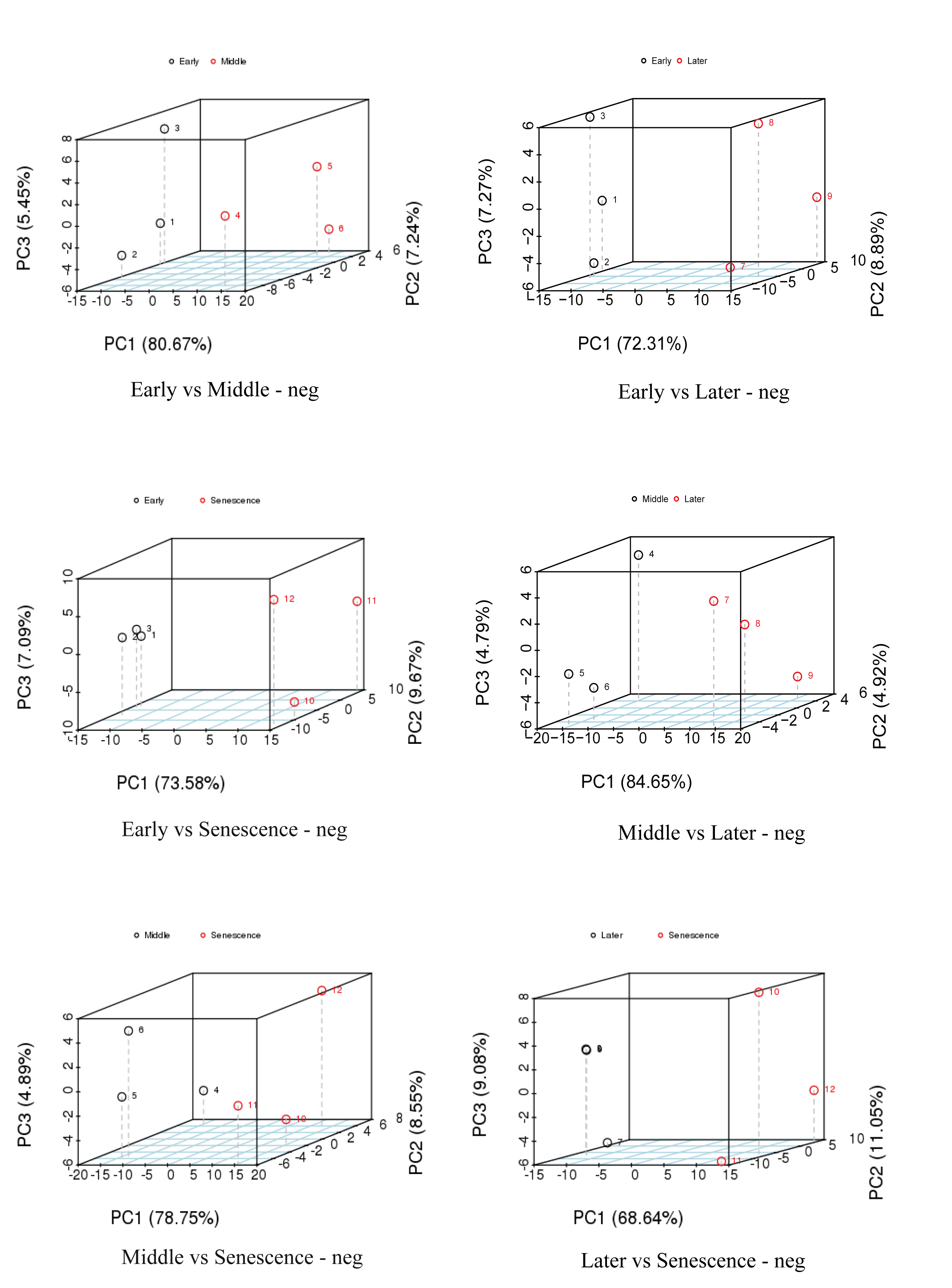

Supplement: Supplementary file 1 [file ijms-25-10355-s001.zip › Supplementary file/S5-PCA_under negtive ion mode.tif]

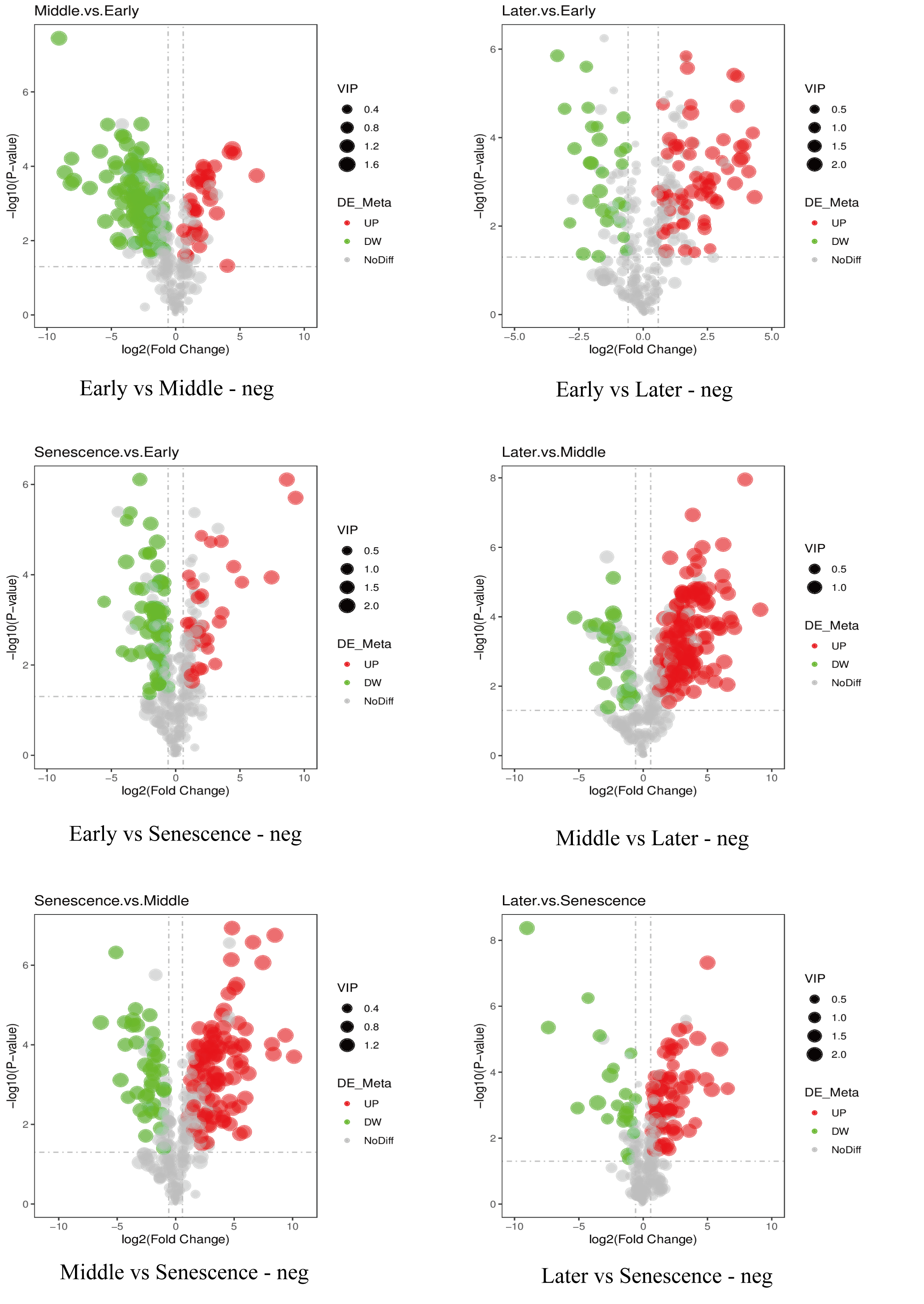

Supplement: Supplementary file 1 [file ijms-25-10355-s001.zip › Supplementary file/S6-Volcano plots_under negtive ion mode.tif]
